# Consumer Perception of the Quality of Lamb and Lamb Confit

**DOI:** 10.3390/foods7050080

**Published:** 2018-05-22

**Authors:** Guillermo Ripoll, Margalida Joy, Begoña Panea

**Affiliations:** Centro de Investigación y Tecnología Agroalimentaria de Aragón (CITA), Instituto Agroalimentario de Aragón—IA2 (CITA-Universidad de Zaragoza), Avda. Montañana 930, 50059 Zaragoza, Spain; mjoy@aragon.es (M.J.); bpanea@aragon.es (B.P.)

**Keywords:** cluster, intrinsic, extrinsic, oil, meat confit, lamb

## Abstract

The patterns of food consumption in general and those of meat, in particular, are constantly changing. These changes are due not only to socioeconomic and cultural trends that affect the whole society but also to the specific lifestyles of consumer groups. Due to the importance of consumer lifestyle, the objectives of this study were (i) to identify the profiles of lamb meat consumers according to their orientation toward convenience, as defined by their eating and cooking habits; (ii) to characterize these profiles according to their socioeconomic characteristics and their preferences regarding the intrinsic and extrinsic quality signals of lamb meat; and (iii) to analyze the willingness to pay for lamb confit. In this study, four types of consumers have been differentiated according to their lifestyles related to lamb consumption. These groups, due to their characteristics, could be called “Gourmet”, “Disinterested”, “Conservative”, and “Basic”. The Gourmet group has characteristics that make it especially interesting to market a product such as lamb confit. However, this group is unaware of this product. Therefore, a possible strategy to expand the commercialization of light lamb and the confit product would be guided marketing to this niche market.

## 1. Introduction

The patterns of food consumption, in general, and those of meat, in particular, are constantly changing. These changes are due not only to socioeconomic and cultural trends that affect the whole society but also to the specific lifestyles of consumer groups, which are increasingly diversified. Lifestyle defines the activities of people in terms of how they spend their time, their interests, their opinions, and their views of themselves and the world around them [1]. This makes the consumer respond differently to everyday stimuli such as consumption. In the field of food marketing research, Brunsø and Grunert [2] established as a methodological framework the food-related lifestyle (FRL) as a mediator between consumer values and their behavior. In this framework, an FRL is composed of five elements or interrelated aspects: how to make purchases, quality aspects for the evaluation of food products, cooking methods, consumption situations, and the reasons for purchase [3]. Bernués, et al. [4] used the methodology of FRL to perform a consumer segmentation regarding fresh lamb meat as convenience food. These convenience foods are increasingly demanded due to the greater incorporation of women into the labor market, the proliferation of small, single-parent or single-person families, individualistic and impulsive consumer behavior, and lack of interest or skill in the kitchen [5]. The four groups of consumers detected were those with a traditional profile, middle-aged people who like traditional foods and exhibit great concern for the intrinsic quality attributes of lamb; people little involved with food but who are aware of its importance and its relationship with health and who are concerned that lamb is easy to cook; the adventurers, with a very open attitude regarding innovation in their diet and a lot of interest in both intrinsic and extrinsic quality of lamb; and finally, the carefree, who are not interested in anything related to food and who are not interested in any particular characteristic of lamb [5]. However, these authors did not conduct any study regarding lamb products with a clear orientation toward convenience purchases, as is the case of light lamb confit and meat that is ready to be consumed after brief and minimal handling. Traditionally, the process of confit is applied to fruits and other products that are cooked in a syrup at low temperature over long cooking times. In recent years, the concept of confit has been extended to cooking in oil at low temperature for long periods, unlike frying, which involves cooking at high oil temperatures for a very short time. An internationally known example is duck confit, in which duck meat confits in its own fat. Light lamb confit is prepared in olive oil at low temperature and is presented as canned in oil. With the production of light lamb confit, which is currently not on the market, the breeders of light lamb would diversify the production and overcome seasonality in the sale of lamb. The diversification of lamb products is interesting due to the constant decrease of fresh lamb in Spain, especially in Aragon [6]. Aragón is an utmost region in the light lamb production and that decrease of meat from light lam consumption is quite harmful. In addition, Ojinegra de Teruel breed is one of the important breeds raised in Aragon. This breed had an early deposition of fat [7], which made it difficult to commercialize it as fresh meat.

Light lamb confit has a flavor somewhat different from lamb cooked by other means and is a product that must simply be heated before serving. As the characteristic flavor of lamb and the difficulty of cooking it are two of the factors that have caused the consumption of lamb to decrease in Spain [8], it is possible that this presentation of light lamb confit, canned with oil, will increase consumption. Currently, the convenience of cooking and consumption is increasingly perceived as a key factor in the marketing of any meat product and, especially for light lamb meat, is closely linked to the manners in which it is cooked and consumed. Therefore, it can be deduced that the future consumption of light lamb meat, or its subsequent replacement by other meats, will depend to a great extent on these two elements: the manners in which it is cooked and consumed. Therefore, the present study has focused on these two aspects to establish consumer orientation toward convenience. This approach is consistent with the “supply, perception and demand for quality” model of food established by [3,4] for the segmentation of consumers.

The objectives of this study were (i) to identify the profiles of light lamb meat consumers according to their orientation toward convenience, as defined by their eating and cooking habits; (ii) to characterize these profiles according to their socioeconomic characteristics and their preferences regarding the intrinsic and extrinsic quality cues of light lamb meat; and (iii) to analyze the willingness to pay for light lamb confit.

## 2. Materials and Methods

An online survey was conducted using forms from Google, Inc. (Menlo Park, CA, USA) during the months of May and June 2014. The geographical scope of the survey was restricted to Spain, ruling out the responses of consumers from other countries. This study was conducted according to the Declaration of Helsinki for studies on human subjects.

The survey consisted of four blocks: (A) socio-demographic variables (sex, age, level of income per capita, level of schooling, etc.); (B) variables related to lifestyle, specifically regarding habits related to eating and cooking; (C) the importance of extrinsic quality attributes of lamb meat; and (D) the importance of the intrinsic quality attributes of lamb meat at the time of purchase, scored according to a 4-point Likert-type scale ((1) not at all important; (2) not very important; (3) fairly important; (4) very important). Blocks B, C and D are shown in Table 1. For the statements in (B) and (C), the respondent had to express his/her degree of agreement or disagreement on a 4-point Likert-type scale ((1) strongly disagree; (2) disagree; (3) agree; (4) strongly agree).

In addition to the questions related to the place of residence and health (“Would you like to lose weight?” and “Is your cholesterol level high?”), respondents were asked about the price they would be willing to pay for a can of light lamb confit for four people that was preserved in three different types of oil (extra virgin olive, virgin olive or sunflower oil). The options were as follows: less than 10 euros, between 10 and 15 euros and more than 15 euros. In the absence of such a product on the market, these proposed prices are based on confit meats of other types of lamb and other meats in general. Due to not having a closed interval as a price reference, the lower and upper ends were left open.

Except for the variable Age, which was continuous, all the questions in the survey were closed and of an ordinal type (blocks B, C and D) or nominal. 

Once the survey was available online, the access link was disseminated via email to both individuals and institutions and groups (housewives, consumers, cultural associations, etc.). Social networks such as Facebook, Twitter and personal blogs were also used. At the end of the two-month duration of the survey period, 659 surveys had been collected, of which 200 surveys were complete and corresponded to Spanish consumers. 

The study of the consumer sample was performed by means of relative frequencies. The analysis of the variables was performed using the *χ*^2^ test, taking a probability less than 0.10 as significant. When one of the cells had a frequency less than five, which makes the use of the *χ*^2^ statistic unadvisable, the likelihood ratio statistic was used at the same probability level. To interpret the pattern of association between the variables studied, the corrected standardized residual between the observed and expected cases within each cell greater than |1.96| was considered. The corresponding percentage associated with these residuals is specified in bold in the tables.

To group consumers into homogeneous groups (clusters or conglomerates), hierarchical cluster analysis was performed using the Ward method. In this analysis, the variables of the B blocks were included, following the theoretical framework of FRL proposed by Brunsø and Grunert [2] and developed by Grunert [9]. The number of clusters or groups of consumers by affinity of their chosen responses was a compromise solution using Ward’s distance from the dendrogram that would maximize the distance between one division of the dendrogram and the next, so as to not obtain a number of clusters too great to be discussed. Subsequently, the relationships between the different groups were analyzed using the test *χ*^2^ test or the likelihood ratio under the conditions discussed above.

## 3. Results

### 3.1. Characterization of the Sample

The data for the general sample are presented in Table 2, Table 3, Table 4, Table 5 and Table 6. The sample (Table 2) had 5% more women, and there was a bias in the level of studies because 50% of the respondents had university studies. Regarding the level of income per capita, less than 25% earned less than €1000/month, and the rest of the sample had income distributed similarly among the three upper strata. Although the survey was initially disseminated throughout Spain and responses were even received from other countries, by not requesting the region of origin of the applicant, we cannot confirm a geographical bias within Spain.

We found that 1.1% of respondents defined themselves as vegetarians (Table 3) and 2.7% held religious beliefs with certain types of food restrictions. Approximately one-third of the sample knew of the Ojinegra de Teruel breed and lamb confit. A large percentage (70.9%) did not know whether they had ever eaten confit, and 12.5% had never tasted it. Only 16.6% answered that they knew of it, of which 72.7% knew it because they had eaten it in a restaurant (Table 3). 

Table 4 presents the degree of agreement with statements that define consumer lifestyle. Most respondents like to eat (96.8%), eat foreign food (72.8%), and prefer informal dinners (70.5%) and variety in food (74.4%), showing great interest in food and an open attitude to different foods and variety. There is a great agreement (greater than 93%) in terms of both the preference to eat at restaurants with friends and family and the importance of dietary planning for family nutrition.

Regarding extrinsic quality attributes (Table 5), the respondents agreed or strongly agreed that lamb meat with some mark of quality is better (78.4%). More than half thought that the lamb of Aragon (51.9%) or organic lamb (57.5%) is better than others, whereas only 32.1% agreed that the Ojinegra de Teruel breed is better than others; 70.4% believe that grass-fed lamb is better, whereas 29.9% think that cereal-fed lamb is best. The price of lamb is very important for 80.1% of respondents, and 68.7% think that lamb is easy to cook.

The importance given to the intrinsic attributes of lamb at the time of purchase is listed in Table 6. The attributes valued as the most important were freshness (95.2%), age (84.5%), low fat content (70.4%) and categorized as light lamb (67.2%). However, 58% of the respondents gave importance to the lamb category, 50.8% preferred lamb to have light-colored meat, and only 38.5% rated the breed as quite or very important at the moment of purchase.

### 3.2. Types of Consumers

Once the general sample was characterized, 4 types of consumers were identified based on their lifestyle related to their cooking and eating habits (Table 2). The first group (CL1) included 82 respondents (41%), group 3 (CL3) included 66 respondents (33%), and group 4 (CL4) accounted for 48 respondents (24%); the second group (CL2) was the minority, representing only 2% of the sample population including 4 respondents. The different groups of consumers did not differ in terms of level of income or place of residence (*p* > 0.10).

Socio-demographic characteristics and lifestyles related to cooking and eating are presented in Table 2 and Table 3, respectively. CL1 was made up of both women and men, most of them with university education. This group was distinct because the percentage of respondents who knew of the Ojinegra de Teruel breed was higher than the general average. In terms of their lifestyle, this group is consumers who love cooking and eating, they live in homes in which everyone cooks, and they spend a lot of time cooking. In addition, they like foreign recipes and variety, and accordingly, they do not believe that lifelong recipes are better. Regarding the importance given to the extrinsic attributes of the quality of lamb meat, this group of consumers exhibited greater disagreement than the general sample regarding which lamb meat with a mark of quality is better, and few strongly disagreed with the ease of cooking lamb. Although this group knew of the Ojinegra de Teruel breed, it did not have a different perception from the other groups regarding this breed having better quality. This response was in accordance with the fact that breed, as an intrinsic attribute of quality, seems to be an unimportant factor. 

CL2 is a minority group, but it has a lifestyle very different from the other groups of consumers; it is formed by women with secondary education or without formal education. One-third of the consumers in this group are vegetarians, and another third have food restrictions because of their religious beliefs. Consumers belonging to this group have a clear disinterest in everything related to cooking and food habits. They do not like foreign food, but they do not think that traditional food recipes are better than modern recipes. They also stand out because they do not like dinners, and they do not cook at home. Compared to the general sample, a significantly greater fraction of this group strongly disagreed that branded lamb is better. All the respondents in this group disagreed strongly with the statement that lamb is easy to cook and that the price of lamb is important. They also considered the appearance of freshness, the age of the lamb and the light color of the meat as significant characteristics.

The third group (CL3) does not like to cook or devote time to cooking, but almost everyone likes to eat; at home, not everyone cooks equally. They do not prefer foreign food because they think that lifelong recipes are best. This group declared mostly not knowing of the Ojinegra de Teruel breed. This group does not stand out from the general sample in terms of the importance assigned to quality attributes, both intrinsic and extrinsic.

CL4 had a higher proportion of men than the other groups. They like to cook but without making changes, and 88.6% agree or strongly agree that traditional recipes are best. This group of consumers also do not like informal dinners. A total of 27.3% of these consumers agreed that lamb meat is easy to cook. Additionally, those that think that price is very important predominate, and there are fewer that disagree with the lamb from Aragon being better than the rest. This group assigned high importance to light-colored meat, although they do not assign more importance to the category (lamb or light lamb) than the other groups or the sample.

### 3.3. Willingness to Pay

**Figure 1:**
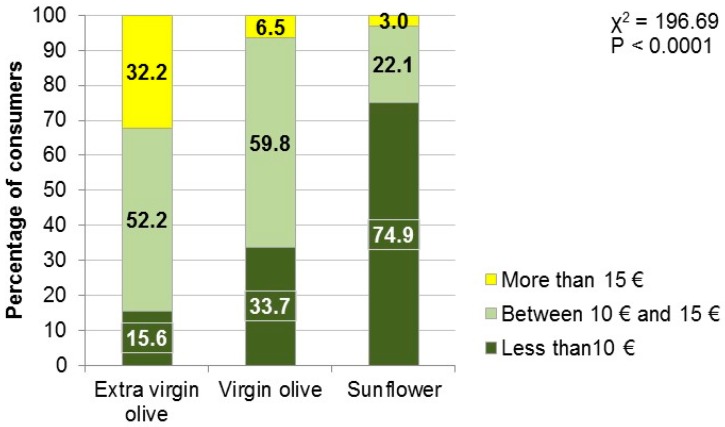
Willingness to pay depending on the type of oil used in the lamb confit.

There was no relationship between the consumer group and the willingness to pay for lamb confit in any type of oil. Of the general sample, almost 32% would pay more than €15 for a can of lamb confit preserved in extra virgin olive oil, and 52% would pay between €10 and €15 (Figure 1). The remaining 15.6% would only pay less than €10. The level of studies, income, sex and all other socio-demographic questions did not have a significant relationship with the willingness to pay for lamb confit (*p* > 0.05) (data not shown). A significant relationship was found (*p* = 0.0362) between consumers with high cholesterol and their willingness to pay. A total of 64.6% of consumers willing to pay between €10 and €15 and 6.3% of those willing to pay less than €10 had high cholesterol. Consumers who would pay more than €15 would do so regardless of whether they had high cholesterol. Consumers who previously knew of lamb confit had a greater willingness (44.4%) to pay more than €15 for lamb confit (*p* = 0.052).

Regarding questions related to consumer habits when cooking and eating, only the frequency with which the consumer eats outside the home (*p* < 0.011) and how important it is to plan meals for family nutrition (*p* < 0.062) affected willingness to pay. Consumers who eat away from home were less willing to pay for lamb confit. Regarding food planning, consumers who agree more about the importance of planning are more willing to pay. In fact, 67.2% of consumers who would pay more than €15 strongly agree about the importance of planning.

The importance assigned by the consumers to most extrinsic quality attributes did not determine the price that he or she would be willing to pay. It did affect the price that would be paid for the lamb to be of Aragonese origin (*p* < 0.01) and for organic lamb (*p* < 0.01); therefore, consumers who do not consider these two attributes important tend to be those who would pay less than €10 for the lamb confit. Consumers who value as important that the lamb they eat is categorized as light lamb (*p* = 0.017) are willing to pay more than €15, whereas consumers who do not give importance to this categorization are only willing to pay up to €10.

## 4. Discussion

According to Cotes [10], the multiple factors that affect the consumption decisions of an individual can be grouped based on his or her demographic characteristics. However, as seen in the results of this work, the behavior of the food consumer does not differ so much based on socio-demographic characteristics, but it does depend on their lifestyles [11]. The so-called psychographic characteristics include all the perceptions or beliefs of the individual, such as beliefs regarding the quality of a brand and propensity to value natural products. Thus, the opinion that each consumer has about the nutritional characteristics or the composition of a product or its safety, brand or price modify decisions at the time of purchase and even the degree of pleasure when it is consumed [12]. In short, consumers relate a group of products to a group of values through a system based on cognitive categories and actions that are embodied in a lifestyle [2].

### 4.1. Types of Consumers

It is possible to define numerous consumer groups; however, when these are framed regarding the lifestyle related to lamb consumption, the number of consumer segments in Spain is usually between three and five [4,13]. Although there are methodological differences in studies, the present work highlights the existence of four groups of differentiated consumers. These groups, due to their characteristics, could be called “Gourmet” (CL1), “Disinterested” (CL2), “Conservative” (CL3) and “Basic” (CL4). When Bredahl and Grunert [14] studied food lifestyles in Spain, they found five segments of consumers. The “Conservative” and “Uninvolved” segments of Bredahl and Grunert [14] are homologous to those found in this work and are called “Conservative” and “Disinterested”, respectively. Consumers in the Conservative group like to cook but without making changes or innovations in the kitchen, which agrees with their opinion that traditional recipes are best. Another segment that the previous authors [4,14] called “Adventurers” would coincide with the gourmet group defined in this work, in that they are very fond of cooking, they use new recipes, and the whole family participates in culinary tasks. The segment called “Rational” by Bredahl and Grunert [14] would be comparable in many of its facets with our “Basic” segment. However, in the case of “Rational”, the social role of food is particularly important, whereas for the “Basic” group, this facet is important but not more so than for other groups. Other studies have also found segments in other countries partially comparable with our results [15,16,17]. Of these recurrent segments in the literature, the one that represents the lowest percentage of the population is the Disinterested, which is formed mostly by women. Buckley, Cowan and McCarthy [15] found a group comparable to the disinterested, consumers who were primarily women who exhibited great individualism in their style of consumption. They do not plan meals or purchases, tend to consume snacks between meals and do not easily accept new products. The fact that women declare that they like exotic foods less agrees with the female gender being positively related to consumer ethnocentrism [18,19]. Thus, consumers with high ethnocentrism prefer to eat and buy products exclusively known and related to their culture. The low consumption of meat is generally a feminine phenomenon [20], and women have a greater number of food restrictions, especially regarding the consumption of red meat [21]. In fact, in a survey in Norway and Sweden, 72.5% of people who consumed little meat were women [22].

### 4.2. Importance of Intrinsic and Extrinsic Attributes

Regarding the importance of the attributes of fresh meat, among the most important according to the results of this study and others are low fat content, freshness and a defined brand or category [4,23,24], all of which are assessed visually. In contrast, breed and organic production are the least valued [4]. Low fat content is an important attribute for all consumers without differences between segments [4]. However, meat with a mark of quality, such as Denomination of Origin or Protected Geographical Indication, can be highly valued or valued very little depending on the consumer group [4], as observed in the present study. The region of origin of the product determines the importance given to some characteristics, even within a certain country. For example, within Spain, consumers in Castilla and León prefer ovine meat from lamb [25], whereas consumers in Aragon [4,26] prefer ovine meat categorized as light lamb. Although it has been seen that breed is not one of the important factors for the consumer, positive value has been given to meat that has been produced in the region where the consumer lives [3,9,26]. However, in our study, the lamb or light lamb category is considered important, but it is not considered that the Ternasco de Aragón PGI (light lamb) or the Ojinegra breed are better than other marks of quality or breeds. As seen in this study, price is a very important factor in general but also for some groups of consumers more than others. Thus, regular lamb consumers perceive higher quality due to marks of quality, diet or ecology. All this information associated with quality makes the price less relevant to the consumer than when there is less information [27].

### 4.3. Willingness to Pay

The process of perception of quality by consumers consists of two phases: the first is based on the perception of extrinsic and intrinsic attributes formed at the point of purchase, and the second is based on the experience formed during the preparation and consumption of the product [28,29]. This is when the expectations formed during the purchase are confirmed or rejected. These two stages are less important when a product is unknown (as may be the case of lamb confit because only a small part of the sample knew of it) or when the consumer does not know whether he or she has tasted it. In this case, the willingness to pay and even the decision to purchase the unknown product are probably based largely on the type of consumer. Thus, an “adventurous” consumer may pay more to try a product that he or she does not know, unlike more conservative or traditional consumers. However, in the scope of this work, there was no differential behavior between groups of consumers for willingness to pay for lamb confit. On the other hand, when the consumer is given information about the quality of the oil used in cooking, which is also a known product, perception of quality and price are positively related [27]. Moreover, although lamb confit is not a known product, olive oil is a well-known and traditional Mediterranean product, and prior knowledge of the product is important when buying food [30,31]. In addition, the appreciation of the different oils includes factors that take into account health [28]. The perception of a food as healthy (as is the case of virgin olive oil), natural, organic or respectful of the environment leads to a greater willingness to pay for the product [31,32], as can be clearly seen in the results of this study. However, when the consumer is not willing to pay a certain price, the conclusion is that the product does not have perceived net value [27], which would be the case of lamb confit preserved in sunflower oil, whose price is limited to a maximum of €15.

## 5. Conclusions

Market segmentation is a necessary requirement to ensure the creation of relationships between products and consumers. In this study, four types of consumers have been differentiated according to their lifestyle related to lamb consumption. One of these groups of consumers (gourmet) has characteristics that make it interesting to market a product such as lamb confit, considering they are unaware of this product. Therefore, a strategy to expand the commercialization of light lamb and the confit product would guide marketing to this niche market. To make the product known, a price strategy could be followed because although price is a clear indicator of perceived quality, when a consumer buys a product repeatedly, he or she gains experience, and the price has a lower weight as a key factor for the purchase. In fact, when the buyer is habitual, he or she values other factors, such as meat, with a mark of quality, even though it has a higher cost associated with it. As there is a direct relationship between the type of oil used in the processing of lamb confit and willingness to pay for the product, a range of products can be made based on this oil and the sale price. In the meat market, there is a range of opportunities that must be exploited with several strategies. In the case of lamb confit, it may be the promotion of the breed as an extrinsic attribute to transmit a higher-quality product.

## Figures and Tables

**Table 1:** Questionnaire about consumer habits when cooking and eating and the importance of extrinsic and intrinsic attributes of lamb meat.

(B) Habits of the consumer at the time of cooking and eating ^1^
I like to cook
I like foreign food
At home, we prefer informal dinners
I like going to restaurants with friends and family
At home, everyone cooks
I spend a lot of time cooking
I like to eat
I like changes in food
Planning food is important for family nutrition
Traditional recipes are best
(C) Importance of extrinsic quality attributes of lamb ^1^
Lamb meat with a mark of quality is better
Lamb is easy to cook
The best lamb is grass-fed
The Ojinegra de Teruel breed is better than others
The price of lamb is very important
The lamb from Aragon is better than others
Organic lamb is better than others
The best lamb is fed cereals
(D) Importance of the intrinsic quality attributes of lamb meat at the time of purchase ^2^
Appearance of freshness
Lamb category
Light lamb category
Light colored meat
Age
Breed
Low fat
(E) Other issues related to lifestyles
Are you vegetarian? ^3^
Do you have any food restrictions for your religion? ^3^
Would you like to lose weight? ^3^
Is your cholesterol level high? ^3^
Do you live in a city? ^3^
Have you heard about the Ojinegra de Teruel breed? ^3^
Have you heard about lamb confit? ^3^
Have you ever eaten lamb confit? ^3^
If you have eaten lamb confit, where did you eat it? ^4^
How much would you be willing to pay for a can for four people of lamb confit preserved in ... ^5^ - Extra virgin olive oil - Olive oil - Sunflower oil

^1^ 4-point Likert scale, Strongly disagree, Disagree, Agree and Strongly agree. ^2^ 4-point Likert-type scale, Not important, A little important, Quite important, Very important. ^3^ Yes/No. ^4^ I cooked it myself, In a restaurant, I bought it made, Other answer. ^5^ Less than 10 euros, between 10 and 15 euros, More than 15 euros.

**Table 2:** Socio-demographic characteristics of the general sample and by consumer group.

	CL1	CL2	CL3	CL4	Total	*χ* ^2^	*p*
Respondents/Percentage of Respondents	82/41%	4/2%	66/33%	48/24%	200		
Sex						6.19	t
Man	41.8	**0.0**	49.2	**59.1**	47.6		
Woman	58.2	**100.0**	50.8	**40.9**	52.4		
Level of schooling						50.87	***
None	0.0	**33.0**	1.6	0.0	1.1		
Primary	0.0	0.0	0.0	2.3	0.5		
Secondary	1.3	**33.3**	3.3	4.5	3.2		
High School	19.0	0.0	21.3	20.5	19.8		
Vocational training	19.0	0.0	26.2	34.1	24.6		
University students	**60.8**	33.3	47.5	38.6	50.8		
Is vegetarian	0.0	**33.3**	1.6	0.0	1.1	31.03	***
Income per capita						17.14	ns
<600 €/month	15.6	**33.3**	5.4	12.2	11.9		
600—1000 €/month	9.1	**33.3**	12.5	22.0	13.6		
1000—1500 €/month	27.3	**33.3**	23.2	12.2	22.6		
1500—2000 €/month	24.7	**0.0**	23.2	31.7	25.4		
>2000 €/month	23.4	**0.0**	35.7	22.6	26.6		
Religious food restrictions	2.5	**33.3**	1.6	2.3	2.7	11.12	*

ns, *p* > 0.10; t, *p* < 0.10; *, *p* < 0.05; ***, *p* < 0.001. Cells in bold had the corrected standardized residual between the observed and expected cases within each cell greater than |1.96|.

**Table 3:** Other questions related to lifestyle.

	CL1	CL2	CL3	CL4	Total	*χ* ^2^	*p*
Respondents/Percentage of Respondents	82/41%	4/2%	66/33%	48/24%	200		
Are you Vegetarian? ^1^	0.0	**33.3**	1.6	0.0	1.1	8.11	*
Do you have any food restrictions for your religion? ^1^	2.5	33.3	1.6	2.3	2.7	3.86	ns
Would you like to lose weight? ^1^	67.1	66.7	76.7	65.9	69.8	2.00	ns
Is your cholesterol level high? ^1^	21.5	66.7	26.2	22.7	24.1	2.91	ns
Do you live in a city? ^1^	81.0	100.0	82.0	77.3	79.4	1.67	ns
Have you heard of the Ojinegra de Teruel breed?	41.8	66.7	21.7	31.8	34.2	7.83	t
Have you heard of lamb confit? ^1^	27.8	33.3	24.6	31.8	29.1	0.71	ns
Have you ever eaten lamb confit? ^1,2^	54.5	100.0	46.7	71.4	16.6	3.06	ns
Where did you eat it? ^3^							
I cooked it myself	30.0	0.0	0.0	0.0	16.0	10.14	ns
In a restaurant	66.7	100.0	85.7	90.0	72.7		
I bought it made	0.0	0.0	14.3	10.0	6.1		
Other answers	13.3	0.0	0.0	0.0	15.2		

^1^ Percentage of affirmative responses. ^2^ The percentage of Do not know/No answer was 70.9%. ^3^ Percentage of respondents who claimed to have eaten lamb confit. Cells in bold had the corrected standardized residual between the observed and expected cases within each cell greater than |1.96|. ns, *p* > 0.10; *, *p* < 0.05.

**Table 4:** Habits of the consumer at the time of cooking and eating for the general sample and by groups of consumers.

	CL1	CL2	CL3	CL4	Total	*χ* ^2^	*p*
Respondents/Percentage of Respondents	82/41%	4/2%	66/33%	48/24%	200		
I like to cook					118.81		ns
Strongly disagree	0.0	66.7	14.8	2.3	6.4		
Disagree	1.3	33.3	47.5	0.0	16.6		
Agree	49.4	0.0	37.7	61.4	47.6		
Strongly agree	49.4	0.0	0.0	36.4	29.4		
I like foreign food					43.95		***
Strongly disagree	0.0	**33.3**	6.6	6.8	4.0		
Disagree	**5.1**	66.7	**37.7**	31.8	23.0		
Agree	**65.8**	0.0	**42.6**	50.0	53.5		
Strongly agree	**29.1**	0.0	13.1	11.4	19.3		
At home, we prefer informal dinners					49.45		***
Strongly disagree	8.9	**100.0**	**1.6**	9.1	8.0		
Disagree	20.3	0.0	13.1	**36.4**	21.4		
Agree	54.4	0.0	62.3	47.7	54.5		
Strongly agree	1.0	0.0	23.0	6.8	16.0		
I like going to restaurants with friends and family					132.77		***
Strongly disagree	0.0	**66.7**	0.0	0.0	1.1		
Disagree	3.8	**33.3**	4.9	6.8	5.3		
Agree	48.1	0.0	57.4	43.2	49.2		
Strongly agree	48.1	0.0	37.7	50.0	44.4		
At home, everyone cooks					48.36		***
Strongly disagree	**8.9**	**33.3**	32.8	**6.8**	16.6		
Disagree	**20.3**	**66.7**	42.6	43.2	33.7		
Agree	43.0	**0.0**	**19.7**	45.5	35.3		
Strongly agree	27.8	**0.0**	**4.9**	**4.5**	14.4		
I spend a lot of time cooking					72.17		***
Strongly disagree	**2.5**	0.0	**29.5**	1.7	11.8		
Disagree	**20.3**	0.0	**59.0**	38.6	36.9		
Agree	**55.7**	66.7	**8.2**	47.7	38.5		
Strongly agree	**21.5**	33.3	**3.3**	9.1	12.8		
I like to eat					143.40		***
Strongly disagree	1.3	**100.0**	0.0	2.3	2.7		
Disagree	0.0	0.0	1.6	0.0	0.5		
Agree	**26.6**	0.0	**72.1**	47.7	46.0		
Strongly agree	**72.2**	0.0	**26.2**	50.0	50.8		
I like changes in food					73.34		***
Strongly disagree	**0.0**	0.0	6.6	**11.4**	4.8		
Disagree	**3.8**	0.0	19.7	**54.5**	20.9		
Agree	43.0	33.3	54.1	31.8	43.9		
Strongly agree	**53.2**	66.7	19.7	**2.3**	30.5		
Planning meals is important for family nutrition					62.06		***
Strongly disagree	2.5	**66.7**	1.6	0.0	2.7		
Disagree	1.3	**33.3**	3.3	2.3	2.7		
Agree	39.2	0.0	39.3	34.1	37.4		
Strongly agree	57.0	0.0	55.7	63.6	57.2		
Traditional recipes are best					77.19		***
Strongly disagree	6.3	**66.7**	9.8	**0.0**	7.0		
Disagree	**51.9**	33.3	**54.1**	**11.4**	42.8		
Agree	41.8	0.0	**27.9**	47.7	38.0		
Strongly agree	**0.0**	0.0	8.2	**40.9**	12.3		

ns, *p* > 0.10; ***, *p* < 0.001. Cells in bold had the corrected standardized residual between the observed and expected cases within each cell greater than |1.96|.

**Table 5:** Importance of the extrinsic attributes of lamb quality.

	CL1	CL2	CL3	CL4	Total	*χ* ^2^	*p*
Respondents/Percentage of Respondents	82/41%	4/2%	66/33%	48/24%	200		
Lamb meat with a mark of quality is better						15.88	t
Strongly disagree	3.8	33.3	5.0	2.3	4.3		
Disagree	25.3	0.0	11.7	11.6	17.3		
Agree	54.4	66.7	70.0	60.5	61.1		
Strongly agree	16.5	0.0	13.3	25.6	17.3		
Lamb is easy to cook						48.19	*
Strongly disagree	**1.3**	**100**	8.3	2.3	4.9		
Disagree	29.1	0	28.3	20.5	26.5		
Agree	55.7	0	50	50	51.9		
Strongly agree	13.9	0	13.3	**27.3**	16.8		
The best lamb is grass-fed						10.58	ns
Strongly disagree	7.6	33.3	5.0	2.3	5.9		
Disagree	20.3	66.7	23.3	27.3	23.7		
Agree	48.1	0.0	48.3	50.0	47.8		
Strongly agree	24.1	0.0	23.3	20.5	22.6		
The Ojinegra de Teruel breed is better than others						13.85	ns
Strongly disagree	8.9	33.3	10.9	9.8	10.1		
Disagree	65.8	33.3	56.4	46.3	57.9		
Agree	22.8	0.0	29.1	34.1	27.0		
Strongly agree	2.5	33.3	3.6	9.8	5.1		
The price of lamb is very important						25.20	**
Strongly disagree	1.3	**33.3**	1.6	2.3	2.2		
Disagree	17.7	33.3	24.6	7.0	17.7		
Agree	55.7	**0.0**	54.1	76.7	59.1		
Strongly agree	25.3	33.3	19.7	14.0	21.0		
The lamb from Aragon is better than others						14.550	ns
Strongly disagree	2.5	0.0	5.3	4.7	3.9		
Disagree	51.9	0.0	47.4	27.9	44.2		
Agree	25.3	50.0	36.8	37.2	32.0		
Strongly agree	20.3	50.0	10.5	30.2	19.9		
Organic lamb is better than others							
Strongly disagree	11.4	33.3	16.7	4.5	11.8	7.72	ns
Disagree	30.4	33.3	23.3	40.9	30.6		
Agree	43.0	33.3	46.7	40.9	43.5		
Strongly agree	15.2	0.0	13.3	13.6	14.0		
The best lamb is fed cereals						9.52	ns
Strongly disagree	11.4	33.3	6.8	11.6	10.3		
Disagree	60.8	0.0	67.8	51.2	59.8		
Agree	25.3	66.7	20.3	32.6	26.1		
Strongly agree	2.5	0.0	5.1	4.7	3.8		

ns, *p* > 0.10; t, *p* < 0.10; *, *p* < 0.05; **, *p* < 0.01. Cells in bold had the corrected standardized residual between the observed and expected cases within each cell greater than |1.96|.

**Table 6:** Importance of the intrinsic quality attributes of lamb meat at the time of purchase.

	CL1	CL2	CL3	CL4	Total	*χ* ^2^	*p*
Respondents/Percentage of Respondents	82/41%	4/2%	66/33%	48/24%	200		
Appearance of freshness						64.06	***
Nothing	0.0	**33.3**	0.0	0.0	0.5		
Little bit	2.5	0.0	6.6	4.5	4.3		
Quite	29.1	33.3	34.4	29.5	31.0		
A lot	68.4	33.3	59.0	65.9	64.2		
Lamb category						10.11	ns
Nothing	13.9	33.3	8.3	6.8	10.8		
Little bit	29.1	0.0	30.0	38.6	31.2		
Quite	34.2	66.7	46.7	29.5	37.6		
A lot	22.8	0.0	15.0	25.0	20.4		
Light lamb category						6.99	ns
Nothing	8.9	33.3	5.0	4.5	7.0		
Little bit	27.8	0.0	28.3	20.5	25.8		
Quite	41.8	33.3	48.3	50.0	45.7		
A lot	21.5	33.3	18.3	25.0	21.5		
Light colored meat						14.03	ns
Nothing	6.3	33.3	1.7	7.0	5.4		
Little bit	44.3	0.0	50.0	37.2	43.8		
Quite	38.0	33.3	41.7	34.9	38.4		
A lot	11.4	33.3	6.7	20.9	12.4		
Age						14.48	ns
Nothing	3.8	33.3	0.0	2.3	2.7		
Little bit	11.4	0.0	13.1	15.9	12.8		
Quite	48.1	33.3	47.5	38.6	45.5		
A lot	36.7	33.3	39.3	43.2	39.0		
Breed							
Nothing	11.4	33.3	**1.6**	6.8	7.5	18.82	*
Little bit	**62.0**	0.0	52.5	45.5	54.0		
Quite	**16.5**	33.3	36.1	38.6	28.3		
A lot	10.1	33.3	9.8	9.1	10.2		
Low fat						11.78	ns
Nothing	5.1	33.3	1.7	2.3	3.8		
Little bit	22.8	0.0	31.7	25.0	25.8		
Quite	51.9	33.3	45.0	56.8	50.5		
A lot	20.3	33.3	21.7	15.9	19.9		

ns, *p* > 0.10; *, *p* < 0.05; ***, *p* < 0.001. Cells in bold had the corrected standardized residual between the observed and expected cases within each cell greater than |1.96|.
